# SGLT2 inhibitors, GLP-1 RAs, and DPP4 inhibitors and the risk of hypomagnesemia in type 2 diabetes: A target trial emulation

**DOI:** 10.1371/journal.pmed.1004968

**Published:** 2026-03-06

**Authors:** Shih-Chieh Shao, Daniel Hsiang-Te Tsai, Albert Tzu-Ming Chuang, Kuan-Hung Liu, Edward Chia-Cheng Lai

**Affiliations:** 1 Department of Pharmacy, Keelung Chang Gung Memorial Hospital, Keelung, Taiwan; 2 School of Pharmacy, Institute of Clinical Pharmacy and Pharmaceutical Sciences, College of Medicine, National Cheng Kung University, Tainan, Taiwan; 3 Population Health Data Center, National Cheng Kung University, Tainan, Taiwan; 4 Institute of Clinical Medicine, College of Medicine, National Cheng Kung University, Tainan, Taiwan; 5 Division of Nephrology, Department of Internal Medicine, National Cheng Kung University Hospital, College of Medicine, National Cheng Kung University, Tainan, Taiwan; Chinese University of Hong Kong, CHINA

## Abstract

**Background:**

Recent studies have suggested potential effects on magnesium homeostasis associated with sodium-glucose cotransporter 2 (SGLT2) inhibitors and glucagon-like peptide-1 receptor agonists (GLP-1 RA). We sought to determine whether these effects lead to a reduced risk of hypomagnesemia among adults with type 2 diabetes in clinical practice.

**Methods and findings:**

We conducted a target trial emulation study using the multi-institutional cohort data from the TriNetX Global Collaborative Network. We compared 1:1 propensity score-matched patients with type 2 diabetes newly initiating SGLT2 inhibitors versus dipeptidyl peptidase-4 (DPP4) inhibitors (*n* = 718,798), GLP-1 RAs versus DPP4 inhibitors (*n* = 623,390), and SGLT2 inhibitors versus GLP-1 RAs (*n* = 702,808) from 2016 to 2024. Propensity scores were estimated using logistic regression models that included baseline covariates such as age, sex, race, comorbidities, and medication and laboratory data. In each comparison, patients receiving DPP4 inhibitors were considered the active-comparator group. The primary outcome was incident hypomagnesemia, defined by clinical diagnosis or serum magnesium <1.80 mg/dL. Patients were followed until the occurrence of an outcome of interest, last clinical visit, death, or the end of database period (March 31, 2025), whichever came first. We found that initiation of SGLT2 inhibitors was associated with a significantly lower risk of hypomagnesemia, compared with both DPP4 inhibitors (Hazard ratio [HR]: 0.80, 95% confidence interval [CI]: 0.79, 0.82; *p* < 0.05) and GLP-1 RAs (HR: 0.92; 95% CI: 0.91, 0.93; *p* < 0.05). Similarly, GLP-1 RA use was associated with a lower risk of hypomagnesemia, compared with DPP4 inhibitors (HR: 0.89; 95% CI: 0.88, 0.91; *p* < 0.05). These associations were consistent across individual agents within each drug class. The main limitation of our study is that residual confounding inherent to retrospective observational research cannot be completely ruled out.

**Conclusions:**

Treatment with SGLT2 inhibitors and GLP-1 RAs was associated with a lower risk of hypomagnesemia, compared with DPP4 inhibitors. These findings suggest that SGLT2 inhibitors and GLP-1 RAs may be preferable options for patients at risk of hypomagnesemia.

## Author summary

**Why was this study done?:**

- Hypomagnesemia affects up to 25% of patients with type 2 diabetes, and it is associated with aggravated diabetes progression and a higher risk of systemic complications.
- Sodium-glucose cotransporter 2 (SGLT2) inhibitors and glucagon-like peptide-1 receptor agonists (GLP-1 RAs) have become an integral part of type 2 diabetes management due to their benefits beyond glucose control, including improvements in cardiorenal function and metabolic outcomes.
- Recent studies have suggested potential effects on magnesium homeostasis associated with SGLT2 inhibitors and GLP-1 RAs. However, it is unknown whether these effects lead to a reduced risk of hypomagnesemia in clinical practice.

**What did the researchers do and find?:**

- Using data from the TriNetX Global Collaborative Network, we found that, compared with dipeptidyl peptidase-4 (DPP4) inhibitors, SGLT2 inhibitors, and GLP-1 RAs were associated with a lower risk of hypomagnesemia in adults with type 2 diabetes.
- In head-to-head comparisons of SGLT2 inhibitors versus GLP-1 RAs, initiation of SGLT2 inhibitors was also associated with a lower risk of hypomagnesemia.
- Consistent findings across individual SGLT2 inhibitors and GLP-1 RAs support potential class effects in maintaining magnesium homeostasis, beyond their established cardiorenal benefits.

**What do these findings mean?:**

- Our findings highlight a potentially important pleiotropic benefit of SGLT2 inhibitors and GLP-1 RAs in attenuating magnesium loss. Clinicians should consider this effect when selecting antihyperglycemic drugs, particularly for people at risk of hypomagnesemia.
- Recognizing and leveraging the mineral-regulating properties of SGLT2 inhibitors and GLP-1 RAs may provide an additional strategy to optimize diabetes management beyond glycemic control.
- Randomized trials are warranted to further establish causal inference, as observational studies are inherently subject to residual confounding despite rigorous design and analytical adjustment.

## Abstract 3

In a target trial emulation, Shih-Chieh Shao and colleagues investigate the risk of hypomagnesemia in type 2 diabetes when treated with GLP-1 RAs or SGLT2i compared to treatment with DDP4 inhibitors, using data from the TriNetX global collaborative network.

## Introduction

Hypomagnesemia, defined as serum magnesium levels below 1.8 mg/dL (0.75 mmol/L), is a common condition in patients with type 2 diabetes, affecting up to 25% of this population—about 10 times more frequently than in the general population [1,2]. This condition is clinically significant, as it is associated with aggravated diabetes progression and a higher risk of systemic complications, such as arrhythmia, which can significantly impact patient health and quality of life. Therefore, strategies to reduce the risk of hypomagnesemia in this group of patients are critically needed.

Sodium-glucose cotransporter 2 (SGLT2) inhibitors have become an integral part of type 2 diabetes management due to their benefits beyond glucose control, including improvements in cardiorenal function and metabolic outcomes [3–11]. Recent research has suggested that SGLT2 inhibition may influence a variety of local and systemic factors, both hormonal and nonhormonal, that could play a role in maintaining magnesium homeostasis [12]. A meta-analysis of 25 randomized trials, including 23 placebo-controlled trials, with a median follow-up of 58.7 weeks, showed that SGLT2 inhibitors increased serum magnesium by a weighted mean difference of 0.07 mmol/L (95% CI: 0.06–0.08), compared with placebo [13]. However, it is unknown whether this increase in magnesium levels observed from clinical trials also leads to a reduced risk of hypomagnesemia in clinical settings. Moreover, most included trials have excluded patients with chronic kidney disease (CKD), thereby limiting generalizability to patients without CKD. Additionally, preliminary evidence suggests that glucagon-like peptide-1 receptor agonists (GLP-1 RAs) reduce urinary magnesium excretion and potentially preserve serum magnesium levels in individuals with type 2 diabetes [14], but large-scale epidemiological data are also lacking. Therefore, further studies on the risks of hypomagnesemia are warranted, especially focusing on active comparisons of different anti-diabetic drug classes pertinent to current practice in a broad range of patients with type 2 diabetes. This study aimed to evaluate the comparative effectiveness of SGLT2 inhibitors, GLP-1 RAs, and dipeptidyl peptidase-4 (DPP4) inhibitors in lowering the risk of hypomagnesemia among patients with type 2 diabetes.

## Methods

### Data source

In this study, we used the TriNetX Global Collaborative Network, a large-scale federated database containing anonymized electronic health records from over 153.5 million patients across more than 128 healthcare organizations (HCOs) in 21 countries, spanning the Americas, Europe, the Middle East, Africa, and the Asia-Pacific region, based on data statistics as of September 2024. HCOs are typically healthcare systems based in academic medical centers or research-oriented nonacademic institutions that agree to share de-identified patient data for research purposes. This platform includes data on patients’ demographics, diagnoses [encoded as International Classification of Diseases, Tenth Revision, Clinical Modification (ICD-10-CM) codes], medications, and procedures. Diagnosis, procedure, and laboratory codes generated by participating HCOs in this network have demonstrated high data quality and completeness. Recent studies have also demonstrated its critical role as an essential data resource for generating real-world evidence for clinical care [15,16]. This study followed the Transparent Reporting of Observational Studies Emulating a Target Trial (TARGET) reporting guidelines (S1 Table) [17,18].

### Specification of the target trial

We designed this observational study to emulate a target trial, defined as a hypothetical pragmatic trial that would directly evaluate the risk of hypomagnesemia among patients with type 2 diabetes initiating SGLT2 inhibitors, DPP4 inhibitors, or GLP-1 RAs. The study outcome of hypomagnesemia was defined as a hypomagnesemia diagnosis (ICD-10-CM: E83.42) or serum magnesium levels less than 1.80 mg/dL [19]. The key components of the target trial are presented in S2 Table.

### Emulation of the target trial

Using multicenter electronic health records from the TriNetX platform, we conducted a target trial emulation study to improve the quality of an observational study investigating the effects of interventions [20,21]. The target trial emulation framework involves designing and analyzing an observational study to replicate the structure and objectives of a hypothetical pragmatic trial addressing the same research question [22,23]. We identified three cohorts of patients who initiated either SGLT2 inhibitors or DPP4 inhibitors (Cohort 1), either GLP-1 RAs or DPP4 inhibitors (Cohort 2), and either SGLT2 inhibitors or GLP-1 RAs (Cohort 3), from 2016 to 2024. Cohort entry (i.e., the index date) was the date of newly receiving a prescription for SGLT2 inhibitors, GLP-1 RAs, or DPP4 inhibitors. DPP4 inhibitors were selected as the comparator due to their widespread use as second- or third-line therapeutic options in the management of type 2 diabetes, similar to SGLT2 inhibitors or GLP-1 RAs. The DPP4 inhibitor group was selected as the comparator to help mitigate potential confounding biases, including those related to treatment indication and variations in disease severity among patients with type 2 diabetes [24–26]. In each cohort, the two treatment groups were independent and mutually exclusive at baseline, meaning that patients had no prior exposure to the drugs of the comparator group before the index date. We restricted the study cohorts to adult patients (aged 18 years or above) with a diagnosis of type 2 diabetes. We excluded patients who had a history of type 1 diabetes or gestational diabetes, alcohol-related disorders, cirrhosis or acute hepatitis, acute or chronic pancreatitis, neoplasms, cachexia, Crohn’s disease, celiac disease, ulcerative colitis, renal tubulopathies, Bartter’s syndrome, intestinal malabsorption, transplanted organ status, gastric bypass surgery or estimated glomerular filtration rate (eGFR) <30 ml/min/1.73m^2^. To ensure incident outcomes, we further excluded patients with a hypomagnesemia diagnosis in the inpatient or outpatient setting before cohort entry. The study design diagram is presented in S1 Fig [27].

We estimated propensity scores using logistic regression models that included the baseline covariates [e.g., age, sex, race, comorbidities (including diabetes complications, cardiovascular diseases, thyroid diseases, liver diseases, CKD, and fluid-related diseases), medications (including anti-diabetes medications, cardiovascular medications, and other drugs potentially affecting serum magnesium hemostasis), and laboratory data (including hemoglobin A1c, eGFR, body mass index and proteinuria)], as listed in Table 1. The disease diagnoses and drug codes used for this study are presented in S3–S6 Tables. To emulate the randomization of the target trial and ensure that the three study cohorts had similar probabilities of treatment assignment, we implemented 1:1 propensity score matching without replacement for each drug comparison (SGLT2 inhibitors versus DPP4 inhibitors, GLP-1 RAs versus DPP4 inhibitors, and SGLT2 inhibitors versus GLP-1 RAs) separately. Matching was performed using a greedy nearest-neighbor algorithm with a caliper width of 0.1 of the standard deviation of the logit of the propensity score to create study cohorts with comparable baseline characteristics [28,29].

**Table 1:** Baseline characteristics of patients with type 2 diabetes initiating SGLT2 inhibitors vs. DPP4 inhibitors, GLP-1 RAs vs. DPP4 inhibitors, and SGLT2 inhibitors vs. GLP-1 RAs, after 1:1 propensity score matching.

	SGLT2 inhibitors vs. DPP4 inhibitors			GLP-1 RAs vs. DPP4 inhibitors			SGLT2 inhibitors vs. GLP-1 RAs		
**SGLT2 inhibitors**	**DPP4 inhibitors**	**ASMD**	**GLP-1 RAs**	**DPP4 inhibitors**	**ASMD**	**SGLT2 inhibitors**	**GLP-1 RAs**	**ASMD**
Age at index, mean years (SD)	62.0 (12.2)	62.1 (12.6)	0.004	60.3 (12.0)	60.6 (12.4)	0.023	59.7 (12.3)	59.6 (12.3)	0.004
Sex, *n* (%)									
Female	164,278 (45.7)	163,723 (45.6)	0.003	152,320 (48.9)	152,893 (49.1)	0.004	165,974 (47.2)	167,168 (47.6)	0.007
Male	187,714 (52.2)	188,421 (52.4)	0.004	151,894 (48.7)	151,319 (48.5)	0.004	176,006 (50.1)	174,844 (49.8)	0.007
Not recorded	7,407 (2.1)	7,255 (2.0)	0.003	7,481 (2.4)	7,483 (2.4)	<0.001	9,424 (2.7)	9,392 (2.7)	0.001
Race, *n* (%)									
White	180,486 (50.2)	182,634 (50.8)	0.012	177,425 (56.9)	179,849 (57.7)	0.016	204,811 (58.3)	202,910 (57.7)	0.011
American Indian or Alaska Native	1,890 (0.5)	1,930 (0.5)	0.002	1,859 (0.6)	1,941 (0.6)	0.003	2,029 (0.6)	2,012 (0.6)	0.001
Black or African American	56,790 (15.8)	56,417 (15.7)	0.003	53,984 (17.3)	55,237 (17.7)	0.011	60,985 (17.4)	60,793 (17.3)	0.001
Asian	25,396 (7.1)	25,556 (7.1)	0.002	17,293 (5.5)	16,968 (5.4)	0.005	17,714 (5.0)	18,028 (5.1)	0.004
Native Hawaiian or Other Pacific Islander	1,419 (0.4)	1,433 (0.4)	0.001	2,311 (0.7)	2,339 (0.8)	0.001	2,839 (0.8)	2,764 (0.8)	0.002
Others	19,129 (5.3)	19,116 (5.3)	<0.001	14,773 (4.7)	15,144 (4.9)	0.006	19,205 (5.5)	19,066 (5.4)	0.002
Not recorded	74,289 (20.7)	72,313 (20.1)	0.014	44,050 (14.1)	40,217 (12.9)	0.036	43,821 (12.5)	45,831 (13.0)	0.017
Diabetes complication, *n* (%)									
Diabetic nephropathy	23,593 (6.6)	23,591 (6.6)	<0.001	19,282 (6.2)	19,237 (6.2)	0.001	24,228 (6.9)	23,580 (6.7)	0.007
Diabetic retinopathy	10,257 (2.9)	10,301 (2.9)	0.001	9,166 (2.9)	9,175 (2.9)	<0.001	11,981 (3.4)	11,804 (3.4)	0.003
Diabetic neuropathy	21,177 (5.9)	21,066 (5.9)	0.001	19,552 (6.3)	19,525 (6.3)	<0.001	24,172 (6.9)	23,923 (6.8)	0.003
Comorbidities, *n* (%)									
Hypertension	124,452 (34.6)	123,914 (34.5)	0.003	115,195 (37.0)	115,206 (37.0)	<0.001	136,659 (38.9)	135,422 (38.5)	0.007
Disorders of lipoprotein metabolism and other lipidaemias	118,068 (32.9)	117,405 (32.7)	0.004	107,023 (34.3)	106,900 (34.3)	0.001	131,205 (37.3)	130,098 (37.0)	0.007
Ischemic heart diseases	39,304 (10.9)	38,895 (10.8)	0.004	29,044 (9.3)	29,168 (9.4)	0.001	36,555 (10.4)	35,652 (10.1)	0.008
Cerebrovascular diseases	15,632 (4.3)	15,348 (4.3)	0.004	11,051 (3.5)	10,938 (3.5)	0.002	12,370 (3.5)	12,037 (3.4)	0.005
Atrioventricular and left bundle-branch block	3,876 (1.1)	3,797 (1.1)	0.002	2,989 (1.0)	3,056 (1.0)	0.002	4,066 (1.2)	3,863 (1.1)	0.005
Other conduction disorders	3,074 (0.9)	2,998 (0.8)	0.002	2,445 (0.8)	2,488 (0.8)	0.002	3,305 (0.9)	3,158 (0.9)	0.004
Paroxysmal tachycardia	2,842 (0.8)	2,836 (0.8)	<0.001	2,485 (0.8)	2,574 (0.8)	0.003	3,638 (1.0)	3,592 (1.0)	0.001
Atrial fibrillation and flutter	14,740 (4.1)	14,426 (4.0)	0.004	10,866 (3.5)	11,262 (3.6)	0.007	14,169 (4.0)	13,794 (3.9)	0.005
Other cardiac arrhythmias	6,972 (1.9)	6,890 (1.9)	0.002	5,530 (1.8)	5,597 (1.8)	0.002	7,373 (2.1)	7,223 (2.1)	0.003
Disorders of thyroid gland	26,154 (7.3)	26,086 (7.3)	0.001	24,761 (7.9)	24,790 (8.0)	<0.001	30,014 (8.5)	29,864 (8.5)	0.002
Liver diseases	9,580 (2.7)	9,442 (2.6)	0.002	9,128 (2.9)	8,743 (2.8)	0.007	11,677 (3.3)	11,750 (3.3)	0.001
Chronic kidney disease	26,321 (7.3)	26,225 (7.3)	0.001	19,756 (6.3)	19,956 (6.4)	0.003	24,033 (6.8)	23,217 (6.6)	0.009
Other chronic obstructive pulmonary disease	11,287 (3.1)	11,234 (3.1)	0.001	9,160 (2.9)	9,389 (3.0)	0.004	10,995 (3.1)	10,863 (3.1)	0.002
Diseases of the musculoskeletal system and connective tissue	92,586 (25.8)	92,413 (25.7)	0.001	85,864 (27.5)	85,449 (27.4)	0.003	103,496 (29.5)	102,785 (29.2)	0.004
Mental, behavioral, and neurodevelopmental disorders	52,741 (14.7)	52,676 (14.7)	0.001	49,783 (16.0)	49,100 (15.8)	0.006	60,380 (17.2)	60,115 (17.1)	0.002
Other disorders of fluid, electrolyte and acid-base balance	14,186 (3.9)	14,177 (3.9)	<0.001	10,562 (3.4)	10,568 (3.4)	<0.001	12,285 (3.5)	11,968 (3.4)	0.005
Anti-diabetes medications, *n* (%)									
Insulin	59,030 (16.4)	58,736 (16.3)	0.002	52,382 (16.8)	52,008 (16.7)	0.003	66,148 (18.8)	66,008 (18.8)	0.001
Metformin	97,255 (27.1)	96,674 (26.9)	0.004	89,547 (28.7)	88,697 (28.5)	0.006	115,220 (32.8)	114,433 (32.6)	0.005
Thiazolidinedione	7,247 (2.0)	7,154 (2.0)	0.002	6,689 (2.1)	6,752 (2.2)	0.001	9,102 (2.6)	8,971 (2.6)	0.002
Sulfonylurea	44,603 (12.4)	44,949 (12.5)	0.003	39,004 (12.5)	39,781 (12.8)	0.008	44,750 (12.7)	44,408 (12.6)	0.003
Acarbose	793 (0.2)	771 (0.2)	0.001	520 (0.2)	495 (0.2)	0.002	690 (0.2)	708 (0.2)	0.001
Other medications, *n* (%)									
Aspirin	36,486 (10.2)	36,436 (10.1)	<0.001	28,491 (9.1)	28,898 (9.3)	0.005	34,843 (9.9)	34,154 (9.7)	0.007
HMG-CoA reductase inhibitors	106,629 (29.7)	105,735 (29.4)	0.005	93,682 (30.1)	93,892 (30.1)	0.001	122,230 (34.8)	120,770 (34.4)	0.009
Angiotensin II receptor blockers	46,537 (12.9)	46,451 (12.9)	0.001	40,316 (12.9)	39,974 (12.8)	0.003	55,251 (15.7)	54,805 (15.6)	0.003
Angiotensin-converting enzyme inhibitors	51,968 (14.5)	51,637 (14.4)	0.003	47,488 (15.2)	47,918 (15.4)	0.004	60,807 (17.3)	60,105 (17.1)	0.005
Diuretics	25,003 (7.0)	24,608 (6.8)	0.004	19,429 (6.2)	19,761 (6.3)	0.004	27,394 (7.8)	26,779 (7.6)	0.007
β-Blocking agents	62,438 (17.4)	62,104 (17.3)	0.002	52,044 (16.7)	52,482 (16.8)	0.004	67,737 (19.3)	66,659 (19.0)	0.008
Calcium channel blockers	48,491 (13.5)	48,507 (13.5)	<0.001	40,350 (12.9)	40,544 (13.0)	0.002	51,970 (14.8)	51,401 (14.6)	0.005
Antimicrobials	84,222 (23.4)	83,977 (23.4)	0.002	76,526 (24.6)	75,963 (24.4)	0.004	90,321 (25.7)	89,980 (25.6)	0.002
Proton-pump inhibitors	40,650 (11.3)	40,455 (11.3)	0.002	36,565 (11.7)	36,316 (11.7)	0.002	44,674 (12.7)	44,359 (12.6)	0.003
Immunosuppressants	4,607 (1.3)	4,593 (1.3)	<0.001	4,182 (1.3)	4,080 (1.3)	0.003	5,574 (1.6)	5,624 (1.6)	0.001
Laxatives	35,498 (9.9)	35,326 (9.8)	0.002	29,413 (9.4)	29,678 (9.5)	0.003	34,728 (9.9)	34,343 (9.8)	0.004
Laboratory results									
Hemoglobin A1c, *n* (%)									
<7%	49,155 (13.7)	49,118 (13.7)	<0.001	45,281 (14.5)	45,098 (14.5)	0.002	57,633 (16.4)	57,141 (16.3)	0.004
≥7%	106,792 (29.7)	106,092 (29.5)	0.004	97,111 (31.2)	95,756 (30.7)	0.009	113,987 (32.4)	113,023 (32.2)	0.006
eGFR, *n* (%)									
30-60 mL/min/1.73 m^2^	30,445 (8.5)	30,396 (8.5)	<0.001	25,786 (8.3)	26,654 (8.6)	0.010	31,557 (9.0)	30,695 (8.7)	0.009
≥60 mL/min/1.73 m^2^	101,125 (28.1)	100,813 (28.1)	0.002	96,707 (31.0)	96,442 (30.9)	0.002	118,842 (33.8)	117,919 (33.6)	0.006
BMI, *n* (%)									
<30 kg/m^2^	60,872 (16.9)	60,833 (16.9)	<0.001	46,234 (14.8)	47,692 (15.3)	0.013	53,967 (15.4)	52,455 (14.9)	0.012
≥30 kg/m^2^	90,377 (25.1)	88,947 (24.7)	0.009	93,382 (30.0)	91,039 (29.2)	0.016	127,149 (36.2)	126,478 (36.0)	0.004
Protein [Mass/volume] in Urine, *n* (%)									
<300 mg/dL	12,409 (3.5)	12,384 (3.4)	<0.001	9,983 (3.2)	9,980 (3.2)	<0.001	12,726 (3.6)	12,451 (3.5)	0.004
≥300 mg/dL	2,339 (0.7)	2,356 (0.7)	0.001	793 (0.3)	754 (0.2)	0.003	2,300 (0.7)	2,194 (0.6)	0.004

Abbreviations: DPP4, dipeptidyl peptidase-4; eGFR, estimated glomerular filtration rate; GLP-1 RA, glucagon-like peptide-1 receptor agonist; SGLT2, sodium-glucose cotransporter 2; ASMD, absolute standardized mean difference.

For missing baseline laboratory data, a “no measurement” category was assigned to account for potential unmeasured factors contributing to the absence of these results, and to ensure that such patients were retained in the matching process [20,21]. The balance of baseline characteristics was assessed using absolute standardized mean differences (ASMDs), with ASMD less than 0.1 indicating a sufficient balance [28,30].

### Statistical analysis

The comparative risks of hypomagnesemia for SGLT2 inhibitors versus DPP4 inhibitors, GLP-1 RAs versus DPP4 inhibitors, and SGLT2 inhibitors versus GLP-1 RAs were evaluated using Cox proportional hazards regression under a fixed-effects model. We estimated hazard ratios (HRs) with 95% confidence intervals (CIs), and also reported absolute risk differences with corresponding 95% CIs. We followed up each eligible patient from the index date until the occurrence of an outcome (e.g., hypomagnesemia), loss to follow-up, death, or the end of the database (e.g., March 31, 2025), whichever occurred first, based on intention-to-treat principles. We used Kaplan–Meier curves to estimate time-to-event probabilities. The statistical codes for propensity score matching and Cox proportional hazard model analysis are presented in S7 Table. Furthermore, we conducted several subgroup analyses according to age group (<60 or ≥60 years), sex (male or female), HbA1c level (<7 or ≥7%), eGFR level (30–60 or ≥60 mL/min/1.73m^2^), CKD diagnosis (yes or no) and individual drug comparisons to examine the result consistency within different subgroups. To examine whether the findings were consistent among patients with albuminuria but with preserved kidney function, we also conducted subgroup analyses in patients with urinary albumin/creatinine ratio >30 mg/g and eGFR ≥ 60 mL/min/1.73 m². Finally, we estimated the HRs separately for distinct time intervals in the first, second, third, fourth, and fifth years of follow-up to examine the variation in HRs over time [31]. We considered a 2-sided *P* < 0.05 as statistically significant.

All statistical analyses in this study, including propensity score matching, Kaplan–Meier curves, and Cox proportional hazard analysis were performed using the built-in functions of the platform. The platform integrates Java 11.0.16 (with Apache Commons Math 3.6.1), R 4.0.2 (with Hmisc 1-1 and Survival 3.2-3), and Python 3.7 (with lifelines 0.22.4, matplotlib 3.5.1, numpy 1.21.5, pandas 1.3.5, scipy 1.7.3, and statsmodels 0.13.2). Specifically, Java 11.0.16 was used to prepare data for input into the statistical packages, mainly R 4.0.2 with Hmisc 1-1 and the Survival package (3.2-3), an open-source language for statistical computing [32]. The Survival 3.2-3 package includes core survival analysis routines, such as Kaplan–Meier and Aalen–Johansen (multi-state) curves, Cox models, and parametric accelerated failure time models. Outcomes were analyzed using the R Survival package (v3.2-3), and the results were validated through comparison with SAS version 9.4 [32]. Within R, the Hmisc 1-1 package was also used to generate summary tables. Propensity scores calculated by logistic regression were estimated in Python 3.7 with NumPy 1.21.5 [33]. Descriptive statistics were post-processed using data analysis packages (Pandas, NumPy) [34,35].

### Individual drugs in the SGLT2 inhibitor and GLP-1 RA classes

To examine potential variations in the risk of hypomagnesemia among individual drugs within the SGLT2 inhibitor and GLP-1 RA classes, we established separate cohorts for canagliflozin, dapagliflozin, empagliflozin, ertugliflozin, albiglutide, dulaglutide, exenatide, liraglutide, lixisenatide, and semaglutide, and compared each with DPP4 inhibitors. Propensity scores were re-estimated, matching was repeated, and effect estimates for the primary outcome were recalculated accordingly.

### Positive and negative control outcome analyses

To ensure the reliability of our findings, we evaluated the risks of incident hyperkalemia (ICD-10-CM: E87.5, or serum potassium ≥5.5 mmol/L) and appendicitis (ICD-10-CM: K35-37) as positive and negative control outcome analyses, respectively, to replicate known and null associations using the same study design [36].

### Sensitivity analysis

To ensure the robustness of our results, we conducted several additional sensitivity analyses. First, we excluded patients with other disorders of fluid, electrolyte, and acid-base balance, since the presentation and other biochemical abnormalities of these conditions may overlap with those of hypomagnesemia. Second, we restricted our outcome definitions by using only serum magnesium levels <1.8 mg/dL. Third, we re-defined our study outcome based solely on the use of intravenous magnesium sulphate or magnesium chloride, which indicated cases of severe hypomagnesemia requiring treatment. Fourth, we excluded patients who had previously undergone serum magnesium testing. In routine clinical practice, serum magnesium measurement is not commonly performed in patients with type 2 diabetes, but it is typically ordered when physicians suspect specific comorbidities or electrolyte imbalances, which may indicate differences in clinical status or symptom severity. This analysis helped provide evidence of our result robustness by minimizing bias related to indication-based testing practices. Finally, we excluded patients who switched to or initiated another drug class during follow-up to assess the robustness of the findings.

### Ethics approval

The data complies with the Health Insurance Portability and Accountability Act and the General Data Protection Regulation. Ethical approval is not required for studies conducted within the TriNetX network analysis platform because the federated system contains only de-identified data, presented as aggregated counts and statistical summaries. Nevertheless, the study protocol was approved by the Institutional Review Board of the Chang Gung Medical Foundation (Approval No. 202401883B0).

## Results

### Baseline characteristics of the study populations

**Fig 1:**
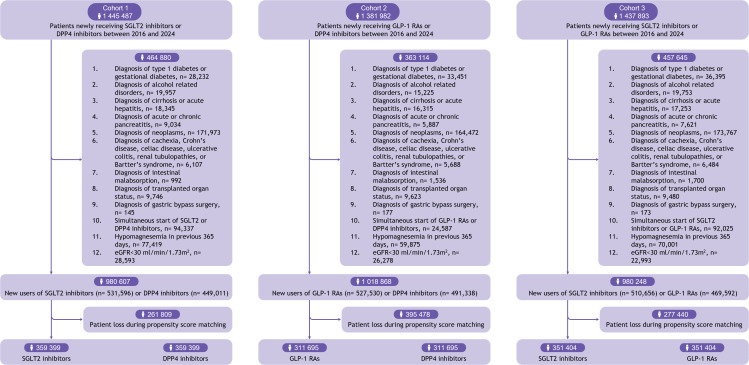
Patient flowchart. Boxes with numbered statements denote the exclusion criteria.

After applying the study inclusion and exclusion criteria (Fig 1), we included 359,399 propensity score-matched pairs in the SGLT2 inhibitor versus DPP4 inhibitor cohort, 311,695 pairs in the GLP-1 RA versus DPP4 inhibitor cohort, and 351,404 matched pairs in the SGLT2 inhibitor versus GLP-1 RA cohort. All three cohorts showed similar distributions of baseline characteristics (e.g., demographics, comorbidities, medications, and relevant laboratory results) with all ASMDs less than 0.1 (Table 1). The propensity score distributions before and after matching are presented in S2 Fig.

In the SGLT2 inhibitor versus DPP4 inhibitor cohort, the mean age of the SGLT2 inhibitor group was 62.0 years, with 45.7% female, 50.2% White, and 7.3% having a history of CKD, all of which were comparable to the DPP4 inhibitor group. Among participants with available laboratory results, the mean HbA1c was 8.3% and the estimated glomerular filtration rate was 80.4 mL/min/1.73 m², which were also similar to the DPP4 inhibitor group. Baseline characteristics were likewise comparable in the GLP-1 RA versus DPP4 inhibitor cohort and in the SGLT2 inhibitor versus GLP-1 RA cohort.

### Risk of hypomagnesemia after initiating SGLT2 inhibitors, GLP-1 RAs and DPP4 inhibitors

**Fig 2:**
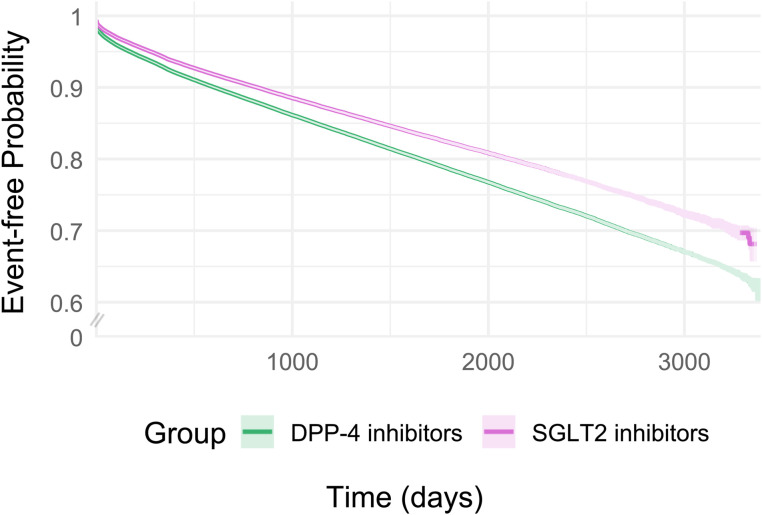
Kaplan–Meier event-free curves for SGLT2 inhibitors vs. DPP4 inhibitors for risk of hypomagnesemia after 1:1 propensity score matching.

**Fig 3:**
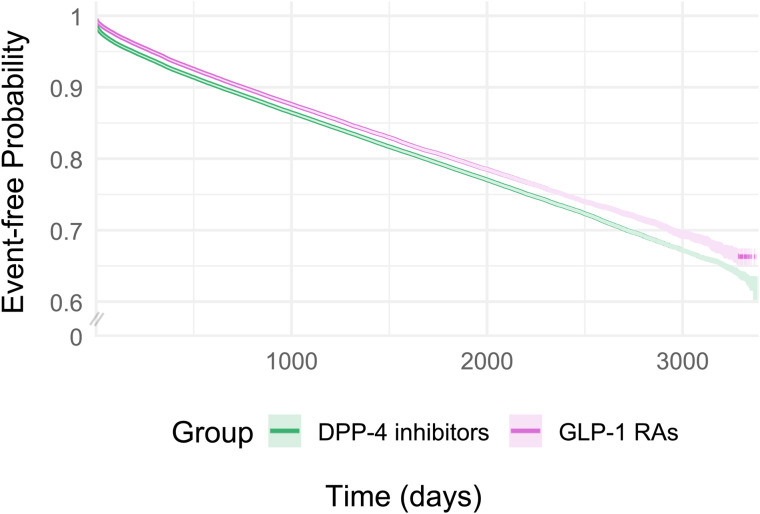
Kaplan–Meier event-free curves for GLP-1 RAs vs. DPP4 inhibitors for risk of hypomagnesemia after 1:1 propensity score matching.

**Fig 4:**
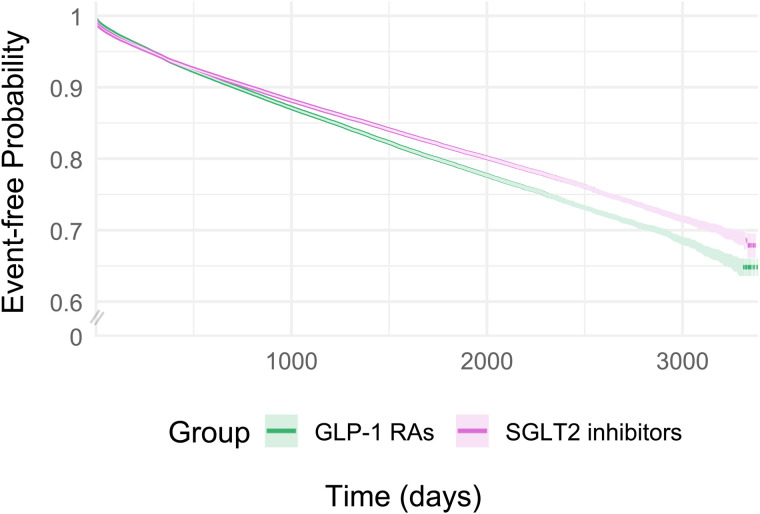
Kaplan–Meier event-free curves for SGLT2 inhibitors vs. GLP-1 RAs for risk of hypomagnesemia after 1:1 propensity score matching.

After a mean follow-up of 34.2 months, we observed 92,352 hypomagnesemia events in the SGLT2 inhibitor versus DPP4 inhibitor cohort. The risk difference was −6.40% (95% CI: −6.55%, −6.25%; *p* < 0.05), and SGLT2 inhibitor use was associated with a lower rate of hypomagnesemia, compared with DPP4 inhibitors (HR: 0.80; 95% CI: 0.79, 0.82; *p* < 0.05). After a mean follow-up of 36.1 months, we observed 84,870 events in the GLP-1 RA versus DPP4 inhibitor cohort. The risk difference was −5.48% (95% CI: −5.65%, −5.31%; *p* < 0.05), and GLP-1 RAs use was associated with a lower rate of hypomagnesemia, compared with DPP4 inhibitors (HR: 0.89; 95% CI: 0.88, 0.91; *p* < 0.05). Finally, after a mean follow-up of 29.7 months, we observed 77,463 events in the SGLT2 inhibitor versus GLP-1 RA cohort. The risk difference was −0.78% (95% CI: −0.93%, −0.64%; *p* < 0.05), and SGLT2 inhibitors were associated with a lower rate of hypomagnesemia than GLP-1 RAs (HR: 0.92; 95% CI: 0.91, 0.93; *p* < 0.05). Figures 2–4 and S8 Table show the cumulative incidence curves and HRs across different time intervals for all three cohorts, respectively.

### Comparative effectiveness of individual SGLT2 inhibitors and GLP-1 RAs versus DPP4 inhibitors

Compared with DPP4 inhibitors, the observed lower risks of hypomagnesemia were consistent for canagliflozin (HR: 0.75; 95% CI: 0.73, 0.78; *p* < 0.05), dapagliflozin (HR: 0.88; 95% CI: 0.87, 0.90; *p* < 0.05), empagliflozin (HR: 0.84; 95% CI: 0.83, 0.85; *p* < 0.05), and ertugliflozin (HR: 0.91; 95% CI: 0.83, 0.997; *p* < 0.05). The results were also consistent for albiglutide (HR: 0.86; 95% CI: 0.75, 0.99; p < 0.05), dulaglutide (HR: 0.94; 95% CI: 0.93, 0.96; *p* < 0.05), exenatide (HR: 0.93; 95% CI: 0.89, 0.97; *p* < 0.05), liraglutide (HR: 0.88; 95% CI: 0.86, 0.89; *p* < 0.05), and semaglutide (HR: 0.84; 95% CI: 0.82, 0.85; *p* < 0.05), but not for lixisenatide (HR: 0.98; 95% CI: 0.90, 1.07; *p* = 0.638) (S9 Table).

### Subgroup and sensitivity analyses

SGLT2 inhibitors and GLP-1 RAs showed lower risks of hypomagnesemia across all subgroups, including different ages (<60 or ≥60 years), sex (female or male), HbA1c levels (<7 or ≥7%), eGFR categories (30−60 or ≥60 mL/min/1.73 m^2^), and presence of CKD (yes or no), compared with DPP4 inhibitors (Table 2). The findings from the subgroup analyses of the SGLT2 inhibitor versus GLP-1 RA cohort were consistent with those in the main analysis (Table 2). The results of the sensitivity analyses were also consistent with the main analysis (S10 Table).

**Table 2:** Results of main and subgroup analyses after 1:1 propensity score matching.

	SGLT2 inhibitors vs. DPP4 inhibitors [Reference]	GLP-1 RAs vs. DPP4 inhibitors [Reference]	SGLT2 inhibitors vs. GLP-1 RAs [Reference]
**HR (95% CI)**	**HR (95% CI)**	**HR (95% CI)**
Main analysis	0.80 (0.79, 0.82)	0.89 (0.88, 0.91)	0.92 (0.91, 0.93)
Age, year			
<60	0.80 (0.79, 0.82)	0.86 (0.85, 0.88)	0.94 (0.92, 0.96)
≥60	0.80 (0.79, 0.82)	0.91 (0.89, 0.92)	0.90 (0.89, 0.92)
Sex			
Male	0.81 (0.79, 0.82)	0.90 (0.88, 0.92)	0.90 (0.88, 0.92)
Female	0.80 (0.78, 0.81)	0.86 (0.85, 0.88)	0.94 (0.92, 0.96)
HbA1c, %			
<7	0.81 (0.80, 0.82)	0.85 (0.83, 0.86)	0.97 (0.95, 0.99)
≥7	0.81 (0.80, 0.82)	0.88 (0.87, 0.90)	0.91 (0.90, 0.92)
eGFR, mL/min/1.73 m^2^			
30–60	0.82 (0.80, 0.83)	0.89 (0.88, 0.90)	0.92 (0.91, 0.94)
≥60	0.81 (0.80, 0.82)	0.88 (0.87, 0.89)	0.92 (0.90, 0.93)
Chronic kidney disease			
Yes	0.80 (0.77, 0.84)	0.96 (0.92, 1.00)	0.86 (0.82, 0.90)
No	0.81 (0.80, 0.82)	0.88 (0.87, 0.89)	0.93 (0.91, 0.94)
UACR >30 mg/g and eGFR ≥ 60 mL/min/1.73 m²	0.89 (0.81, 0.98)	0.86 (0.69, 1.06)	0.89 (0.81, 0.98)

Abbreviations: CI, confidence intervals; DPP4, dipeptidyl peptidase-4; eGFR, estimated glomerular filtration rate; GLP-1 RA, glucagon-like peptide-1 receptor agonist; HR, hazard ratio; SGLT2, sodium-glucose cotransporter 2; UACR, urinary albumin/creatinine ratio.

### Positive and negative control outcome analyses

Representing a known association, the initiation of SGLT2 inhibitors was associated with a significantly lower risk of incident hyperkalemia, compared with both DPP4 inhibitors (HR: 0.84; 95% CI: 0.82, 0.86; *P* < 0.05) and GLP-1 RAs (HR: 0.96; 95% CI: 0.94, 0.98; *P* < 0.05). Similarly, GLP-1 RA use was associated with a lower risk of hyperkalemia, compared with DPP4 inhibitors (HR: 0.91; 95% CI: 0.89, 0.93; *P* < 0.05) (S10 Table). Furthermore, we observed no significant risk difference in incident appendicitis across the three cohort comparisons (HR: 1.03, 95% CI: 0.93, 1.14; *P* = 0.602 for SGLT2 inhibitors versus DPP4 inhibitors; HR: 1.07, 95% CI: 0.96; 1.19; *P* = 0.594 for GLP-1 RAs versus DPP4 inhibitors; HR: 0.97; 95% CI: 0.88, 1.08; *P* = 0.229 for SGLT2 inhibitors versus GLP-1 RAs) (S10 Table).

## Discussion

In this target trial emulation study, our findings suggested that, compared with DPP4 inhibitors, both SGLT2 inhibitors and GLP-1 RAs were associated with reductions in the risk of hypomagnesemia in adults with type 2 diabetes. Notably, the consistent results across a range of individual SGLT2 inhibitors (canagliflozin, dapagliflozin, empagliflozin, and ertugliflozin) and GLP-1 receptor agonists (albiglutide, dulaglutide, exenatide, liraglutide, and semaglutide) underscore the potential class benefits of SGLT2 inhibitors and GLP-1 RAs in maintaining magnesium homeostasis beyond their cardiorenal advantages. In the head-to-head comparisons of SGLT2 inhibitors versus GLP-1 RAs, we observed an additional reduction in the risk of hypomagnesemia after initiation of SGLT2 inhibitors. These results provide evidence of the real-world comparative effectiveness with regard to hypomagnesemia of the three most commonly used second-line antihyperglycemic drug classes, and could guide the choice of these drug classes in clinical practice.

Compared with previous studies, our investigation addressed several key knowledge gaps in existing literature. First, previous studies indicated increases of serum magnesium level by 0.07 mmol/L after SGLT2 inhibitor use in short-term treatment (<1 year) [13]. Our study extended these results to the reduction in risk of hypomagnesemia, as defined by clinical diagnosis or serum magnesium <1.80 mg/dL. Second, two previous studies excluded patients with moderate to severe CKD, potentially limiting the generalizability of their results as regards patients with poor renal functions [37,38]. Our study included a diverse population spanning a wide range of renal functions, and our findings from the subgroup analyses (e.g., eGFR 30−60 or ≥60 mL/min/1.73m^2^, and with or without CKD diagnosis) indicated consistent reductions in the risk of hypomagnesemia in patients receiving SGLT2 inhibitors who had different renal functions at baseline. Finally, previous reports have indicated that GLP-1 RAs potentially reduce magnesium excretion [14], but recent case reports have suggested that the prominent gastrointestinal symptoms from GLP-1 RAs increased the risk of hypomagnesemia [39,40]. Our study provides evidence that treatment with GLP-1 RAs is associated with a reduced risk of hypomagnesemia, possibly because the benefits of improved glycemic control outweigh the gastrointestinal effects on magnesium homeostasis.

SGLT2 inhibitors and GLP-1 RAs appear to mitigate the risk of hypomagnesemia in patients with type 2 diabetes, and some potential mechanisms of this pleiotropic effect on magnesium homeostasis have been proposed. Driven by upregulated transient receptor potential melastatin 6 (TRPM6) channels and elevated uromodulin expression according to proteomic analyses from the EMPEROR-Reduced and EMPEROR-Preserved trials [41,42], SGLT2 inhibitors have been found to enhance magnesium reabsorption in the distal convoluted tubule. Also, osmotic diuresis and mild volume contraction during use of SGLT2 inhibitors can transiently increase serum magnesium and trigger hormonal shifts (e.g., heightened glucagon and vasopressin) that boost renal magnesium reclamation [13,43]. Finally, SGLT2 inhibitor treatment improves insulin sensitivity, which protects against magnesium loss by reducing intracellular shifts, mitigating renal hyperfiltration, and enhancing TRPM6-mediated magnesium uptake in the distal convoluted tubule. Additionally, GLP-1 may have regulatory effects on intestinal magnesium absorption via modulation of hormonal or autonomic pathways [44–50]. The beneficial effects of GLP-1 RAs on magnesium homeostasis are likely to be partly indirect, mediated through improvements in hyperglycemia, insulin resistance, and other components of the metabolic syndrome [49,51–53]. Better glycemic control and reduced insulin resistance may lessen urinary magnesium wasting and enhance renal magnesium retention, while weight loss and overall metabolic improvements further support the normalization of magnesium balance [54,55]. Of note, compared with SGLT2 inhibitors, GLP-1 RAs were associated with a greater reduction in the risk of hypomagnesemia during the first year of follow-up, probably reflecting their more pronounced early effects on glucose control and weight reduction [11], which may have contributed to an initial improvement in magnesium balance. Together, these actions may explain the observed preservation of serum magnesium concentrations during treatment with either class of agents.

Our drug-specific HRs were directionally consistent with a class effect of SGLT2 inhibitors on serum magnesium, although the magnitudes varied. Prior evidence indicates that SGLT2 inhibitors generally increase serum Mg^2+^ concentrations, with drug-level differences plausibly related to varying SGLT2 and SGLT1 selectivity [56–58]. Post-hoc and meta-analytic data suggest that canagliflozin may produce greater mean increases in serum Mg^2+^ and higher rates of normalization among patients with hypomagnesemia, whereas ertugliflozin shows smaller average increases, consistent with our observed rank order [13,59–61]. Nonetheless, our HRs represent comparative effectiveness within this cohort and should not be interpreted as evidence of direct pharmacological superiority among the drugs.

Hypomagnesemia is frequently under-recognized in patients with type 2 diabetes [51,62,63], despite its association with increased cardiovascular risk, insulin resistance, and poor glycemic controls [51,63–65]. Our findings highlight a potentially important pleiotropic benefit of SGLT2 inhibitors and GLP-1 RAs, in that they attenuate magnesium loss. By improving magnesium homeostasis, these agents may contribute to broader metabolic stability and reduce arrhythmic or vascular complications [66]. However, these hypothetical mechanisms require further validation. Clinicians should be aware of this effect when selecting antihyperglycemic therapy, especially in patients at risk of hypomagnesemia. Understanding and leveraging the mineral-regulating properties of SGLT2 inhibitors and GLP-1 RAs should be recognized as a way to further optimize diabetes care beyond glycemic controls.

The large sample size constituted a major strength of this study, permitting detailed evaluation of both important subpopulations and individual drugs by using target trial emulation design with three comparison cohorts (SGLT2 inhibitors versus DPP4 inhibitors, GLP-1 RAs versus DPP4 inhibitors and SGLT2 inhibitors versus GLP-1 RAs), thus giving evidence of the real-world comparative effectiveness with regard to hypomagnesemia of the three most commonly used second-line antihyperglycemic drug classes. However, our study has several limitations. First, we matched a wide range of potential confounders, including kidney function and glycemic levels, in the propensity score model to reduce confounding and selection bias, but residual confounding inherent to retrospective observational research could not be totally ruled out. For example, we did not include any data on dietary magnesium intake and over-the-counter drugs, such as vitamins and mineral supplements paid for by patients. In addition, some patients may have been lost to follow-up or had missing data during the study period, especially given our study’s relatively long follow-up (around 128 weeks). However, any effect from such issues should be evenly distributed across groups under the new-user and active-comparator design used in this study [67]. Moreover, the findings from our positive (hyperkalemia) and negative (appendicitis) control outcome analyses indicated that the presence of unmeasured systematic bias was unlikely, thus supporting the study’s internal validity. Third, our subgroup analyses by individual SGLT2 inhibitors and GLP-1 RAs demonstrated consistent associations with reduced hypomagnesemia risk, though with varying effect sizes. Further head-to-head comparative studies are warranted to clarify the differential risks within each drug class. Fourth, the validity of the ICD-10-CM hypomagnesemia codes in the TriNetX database has not been investigated before, so misclassification of outcomes may have occurred. To increase the validity of our outcome definitions, we also included objective laboratory parameters (serum magnesium <1.80 mg/dL) in the study outcome definition, further reducing the likelihood of misclassification bias in our results. Fifth, we were unable to perform a competing risk analysis, which would have more appropriately accounted for events such as mortality that could influence our primary outcomes, as this function is not supported by the TriNetX platform. However, based on the findings from our negative control outcome analysis (appendicitis), we believe that the impact of death on our primary outcomes was likely minimal. Finally, our observed effect sizes should only be interpreted as intention-to-treat principles with the goal of influencing the selection of medical therapy for well-defined groups of patients [68].

In this target trial emulation study, patients with type 2 diabetes who initiated treatment with SGLT2 inhibitors or GLP-1 RAs showed a reduced risk of hypomagnesemia, compared with similar patients who initiated treatment with DPP4 inhibitors. SGLT2 inhibitors and GLP-1 RAs may be considered to reduce the risk of hypomagnesemia in a broad range of patients with type 2 diabetes.

### Ethics approval and consent to participate

As the data were routinely collected and fully anonymized, patient consent was not required. The study protocol was approved by the institutional review board of Taiwan’s Chang Gung Medical Foundation (No: 202401883B0).

## Supporting information

S1 Fig Study design diagram. (TIF)

S2 Fig Propensity score distributions. (TIF)

S1 Table Checklist for the TARGET guidelines. (DOCX)

S2 Table Specification and emulation of a target trial evaluating the effect of SGLT2 inhibitors, GLP-1 RAs, and DPP4 inhibitors on the risk of hypomagnesemia events using the TriNetX Global Collaborative Network. (DOCX)

S3 Table Disease diagnosis codes for the exclusion criteria. (DOCX)

S4 Table Codes to identify the exposure or comparator drugs. (DOCX)

S5 Table Disease diagnosis codes to identify baseline comorbidities. (DOCX)

S6 Table Codes to identify comedications. (DOCX)

S7 Table R code for running propensity score matching and Cox proportional hazards model analysis. (DOCX)

S8 Table Hypomagnesemia risk across different follow-up time intervals after 1:1 propensity score matching. (DOCX)

S9 Table Hypomagnesemia risk by individual drugs after 1:1 propensity score matching. (DOCX)

S10 Table Results of sensitivity analyses. (DOCX)

## Abbreviations

ASMDs: absolute standardized mean differences
CIs: confidence intervals
CKD: chronic kidney disease
DPP4: dipeptidyl peptidase-4
eGFR: estimated glomerular filtration rate
GLP-1 RAs: glucagon-like peptide-1 receptor agonists
HCOs: healthcare organizations
HRs: hazard ratios
ICD-10-CM: International Classification of Diseases, Tenth Revision, Clinical Modification
SGLT2: sodium-glucose cotransporter-2
TARGET: Transparent Reporting of Observational Studies Emulating a Target Trial
TRPM6: transient receptor potential melastatin 6.
