# Supplementary figures and images for: SGLT2 inhibitors, GLP-1 RAs, and DPP4 inhibitors and the risk of hypomagnesemia in type 2 diabetes: A target trial emulation
Source: PLoS Med. 2026 Mar 6;23(3):e1004968. doi: 10.1371/journal.pmed.1004968 (PMC12987583; doi:10.1371/journal.pmed.1004968)

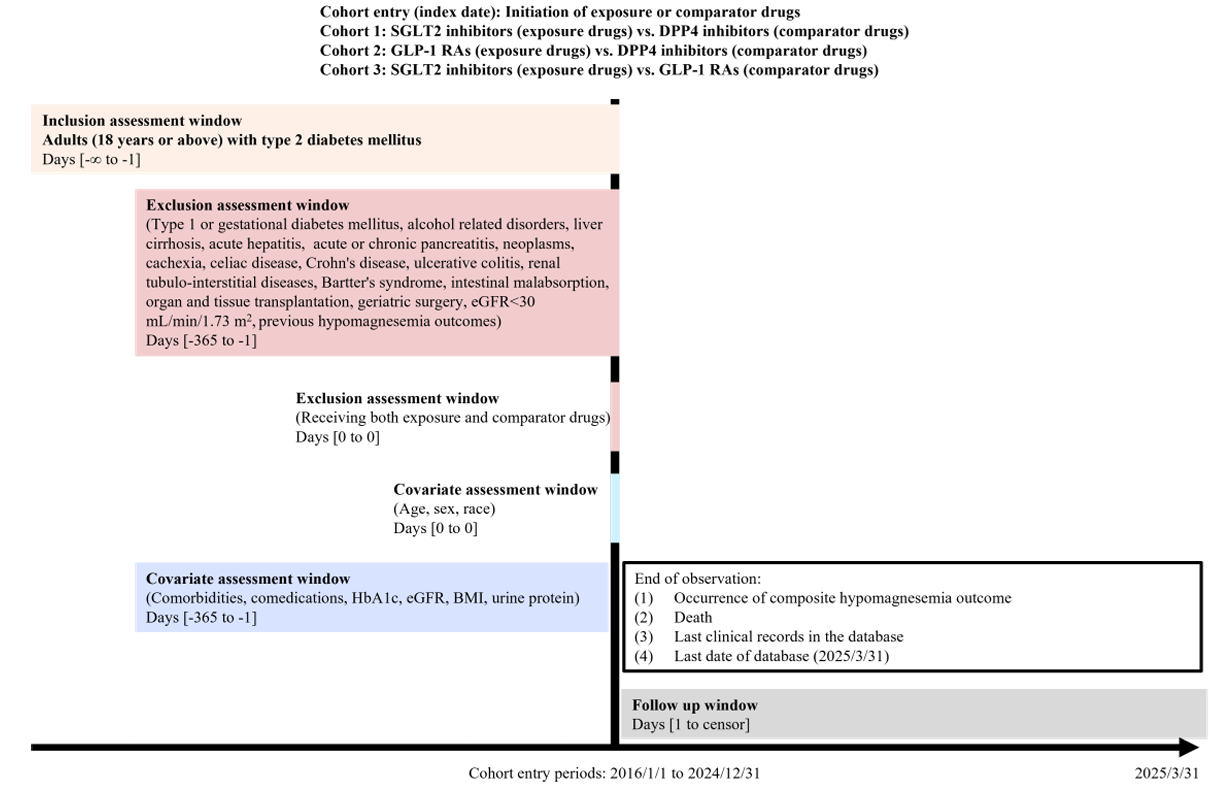

Supplement: S1 Fig — (TIF) [file pmed.1004968.s001.tif]

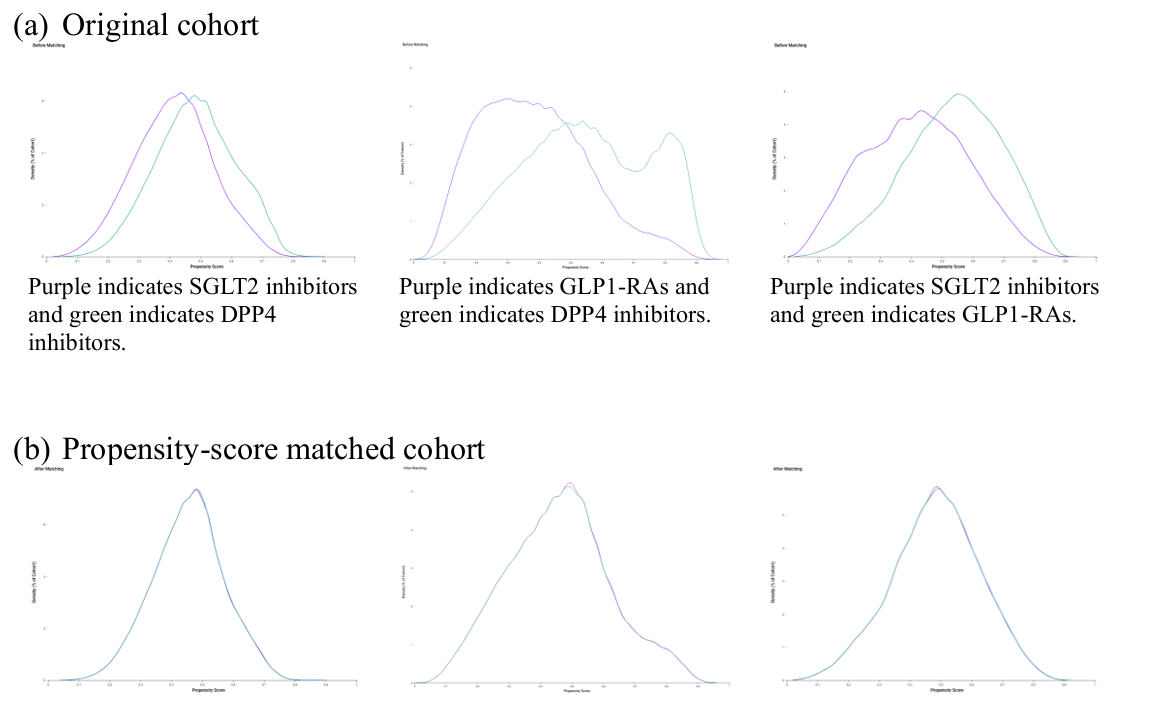

Supplement: S2 Fig — (TIF) [file pmed.1004968.s002.tif]
