# Supplementary material for: SGLT2 inhibitors, GLP-1 RAs, and DPP4 inhibitors and the risk of hypomagnesemia in type 2 diabetes: A target trial emulation
Source: PLoS Med. 2026 Mar 6;23(3):e1004968. doi: 10.1371/journal.pmed.1004968 (PMC12987583; doi:10.1371/journal.pmed.1004968)
Supplement: S2 Table — (DOCX) [file pmed.1004968.s004.docx]

**S2 Table.** Specification and emulation of a target trial evaluating the effect of SGLT2 inhibitors, GLP-1 RAs and DPP4 inhibitors on the risk of hypomagnesemia events using the TriNetX Global Collaborative Network.

| Protocol component | Target trial | Emulated trial using real-world data |
| --- | --- | --- |
| Study aim | To evaluate the long-term risk of hypomagnesemia across different treatment classes. | Same. |
| Study design | Multicenter, parallel-group, randomized trial with open-label, pragmatic conditions. | Retrospective cohort study emulating a target trial through:   1. Data source: TriNetX Global Collaborative Network (electronic health records from >140 healthcare organizations), 2016-2024. 2. New-user and active-comparator design:   Patients newly initiating SGLT2 inhibitors, GLP-1 RAs, or DPP4 inhibitors were included.   1. 1:1 propensity-score matching to balance the patient characteristics. |
| Eligibility  criteria  [Inclusion] | Adult participants (≥18 years) with type 2 diabetes who initiated SGLT2 inhibitors, GLP-1 RAs, or DPP4 inhibitors without prior hypomagnesemia events. | Same. |
| Eligibility criteria  [Exclusion] | 1. Diagnosis of type 1 diabetes or gestational diabetes 2. Diagnosis of alcohol related disorders 3. Diagnosis of cirrhosis or acute hepatitis 4. Diagnosis of acute or chronic pancreatitis 5. Diagnosis of neoplasms 6. Diagnosis of cachexia, Crohn’s disease, celiac disease, ulcerative colitis, renal tubulopathies, or Bartter’s syndrome 7. Diagnosis of intestinal malabsorption 8. Diagnosis of transplanted organ status 9. Diagnosis of gastric bypass surgery 10. Simultaneous start of two treatment classes 11. Previous hypomagnesemia events 12. eGFR<30 ml/min/1.73m^2^ | Same. |
| Treatment strategies | Eligible participants are assigned to one of three different comparison cohorts:  (1) SGLT2 inhibitors vs. DPP4 inhibitors  (2) GLP-1 RAs vs. DPP4 inhibitors  (3) SGLT2 inhibitors vs. GLP-1 RAs | Same. |
| Treatment assignment | 1:1 random assignment to either treatment group within different comparisons. | 1:1 propensity-score matching conditioned on prespecified baseline covariates, including age, sex, race, comorbidities, medication and laboratory data within three pairwise comparisons. |
| Outcomes | Incident hypomagnesemia events. | Same. However, we used hypomagnesemia diagnoses (ICD-10-CM: E83.42) or serum magnesium levels less than 1.80 mg/dL to identify study outcomes. |
| Follow-up | We followed participants from the initiation of SGLT2 inhibitors, DPP4 inhibitors or GLP-1 RAs to the occurrence of an outcome, loss to follow-up, death, or the end of the database (i.e., March 31, 2025) | Same. |
| Causal contrasts | Intention-to-treat approach. | Same. |
| Statistical analysis | 1. Cox proportional hazards models for time-to-event outcomes. 2. Pre-specified subgroup and sensitivity analyses. | Same. However, we additionally conducted positive (hyperkalemia) and negative (appendicitis) control outcomes to determine the study’s internal validity. |

DPP4: dipeptidyl peptidase-4, eGFR: estimated glomerular filtration rate, GLP-1 RAs: glucagon-like peptide-1 receptor agonists, ICD-10-CM: International Classification of Diseases, Tenth Revision, Clinical Modification, SGLT2: sodium-glucose cotransporter-2.
