# Supplementary material for: SGLT2 inhibitors, GLP-1 RAs, and DPP4 inhibitors and the risk of hypomagnesemia in type 2 diabetes: A target trial emulation
Source: PLoS Med. 2026 Mar 6;23(3):e1004968. doi: 10.1371/journal.pmed.1004968 (PMC12987583; doi:10.1371/journal.pmed.1004968)
Supplement: S3 Table — (DOCX) [file pmed.1004968.s005.docx]

**Supplementary Table 3**. Disease diagnosis codes for the exclusion criteria.

| Disease | ICD-10-CM |
| --- | --- |
| Type 1 diabetes mellitus | E10 |
| Gestational diabetes mellitus | O24.4 |
| Alcohol related disorders | F10 |
| Primary biliary cirrhosis | K74.3 |
| Secondary biliary cirrhosis | K74.4 |
| Biliary cirrhosis, unspecified | K74.5 |
| Other and unspecified cirrhosis of the liver | K74.6 |
| Acute hepatitis A | B15 |
| Acute hepatitis B | B16 |
| Acute pancreatitis | K85 |
| Alcohol-induced chronic pancreatitis | K86.0 |
| Other chronic pancreatitis | K86.1 |
| Neoplasms | C00–D49 |
| Cachexia | R64 |
| Celiac disease | K90.0 |
| Crohn’s disease [regional enteritis] | K50 |
| Ulcerative colitis | K51 |
| Other specified renal tubulo-interstitial diseases | N15.8 |
| Bartter’s syndrome | E26.81 |
| Intestinal malabsorption | K90 |
| Transplanted organ and tissue status | Z94 |
| Hypomagnesemia | E83.42 |

ICD-10-CM: International Classification of Diseases, 10th Revision, Clinical Modification
