# Supplementary material for: SGLT2 inhibitors, GLP-1 RAs, and DPP4 inhibitors and the risk of hypomagnesemia in type 2 diabetes: A target trial emulation
Source: PLoS Med. 2026 Mar 6;23(3):e1004968. doi: 10.1371/journal.pmed.1004968 (PMC12987583; doi:10.1371/journal.pmed.1004968)
Supplement: S4 Table — (DOCX) [file pmed.1004968.s006.docx]

**S4 Table.** Codes to identify the exposure or comparator drugs.

| Drugs | | RxNORM |
| --- | --- | --- |
| SGLT2 inhibitors | Empagliflozin | 1545653 |
|  | Dapagliflozin | 1488564 |
|  | Canagliflozin | 1373458 |
|  | Ertugliflozin | 1992672 |
| DPP4 inhibitors | Linagliptin | 1100699 |
|  | Sitagliptin | 593411 |
|  | Saxagliptin | 857974 |
|  | Alogliptin | 1368001 |
|  | Vildagliptin | 596554 |
| GLP-1 RAs | Liraglutide | 475968 |
|  | Dulaglutide | 1551291 |
|  | Semaglutide | 1991302 |
|  | Lixisenatide | 1440051 |
|  | Exenatide | 60548 |
|  | Albiglutide | 1534763 |

DPP4: dipeptidyl peptidase-4, GLP-1 RAs: glucagon-like peptide-1 receptor agonists, SGLT2: sodium-glucose cotransporter-2
