# Supplementary material for: SGLT2 inhibitors, GLP-1 RAs, and DPP4 inhibitors and the risk of hypomagnesemia in type 2 diabetes: A target trial emulation
Source: PLoS Med. 2026 Mar 6;23(3):e1004968. doi: 10.1371/journal.pmed.1004968 (PMC12987583; doi:10.1371/journal.pmed.1004968)
Supplement: S5 Table — (DOCX) [file pmed.1004968.s007.docx]

**S5 Table.** Disease diagnosis codes to identify baseline comorbidities.

| Comorbidity | ICD-10-CM |
| --- | --- |
| Disorders of the thyroid gland | E00–E07 |
| Type 2 diabetes mellitus with kidney complications | E11.2 |
| Type 2 diabetes mellitus with ophthalmic complications | E11.3 |
| Type 2 diabetes mellitus with neurological complications | E11.4 |
| Disorders of lipoprotein metabolism and other lipidemias | E78 |
| Other disorders of fluid, electrolyte and acid-base balance | E87 |
| Mental, behavioral and neurodevelopmental disorders | F01–F99 |
| Other chronic obstructive pulmonary disease | J44 |
| Diseases of the liver | K70–K77 |
| Diseases of the musculoskeletal system and connective tissues | M00–M99 |
| Chronic kidney disease | N18 |
| Essential (primary) hypertension | I10 |
| Ischemic heart diseases | I20–I25 |
| Atrioventricular and left bundle-branch block | I44 |
| Other conduction disorders | I45 |
| Paroxysmal tachycardia | I47 |
| Atrial fibrillation and flutter | I48 |
| Other cardiac arrhythmias | I49 |
| Cerebrovascular diseases | I60–I69 |

ICD-10-CM: International Classification of Diseases, 10th Revision, Clinical Modification
