# Supplementary material for: SGLT2 inhibitors, GLP-1 RAs, and DPP4 inhibitors and the risk of hypomagnesemia in type 2 diabetes: A target trial emulation
Source: PLoS Med. 2026 Mar 6;23(3):e1004968. doi: 10.1371/journal.pmed.1004968 (PMC12987583; doi:10.1371/journal.pmed.1004968)
Supplement: S6 Table — (DOCX) [file pmed.1004968.s008.docx]

**S6 Table.** Codes to identify comedications.

| Drugs | | RxNORM/ATC |
| --- | --- | --- |
| Diabetes medications | Insulin | HS501 |
|  | Metformin | 6809 |
|  | Thiazolidinediones | A10BG |
|  | Sulfonylureas | A10BB |
|  | Acarbose | 16681 |
| Others | Aspirin | 1191 |
|  | HMG CoA Reductase Inhibitors | C10AA |
|  | Angiotensin II Inhibitor | CV805 |
|  | Ace Inhibitors | CV800 |
|  | Loop Diuretics | CV702 |
|  | Beta Blockers/Related | CV100 |
|  | Calcium Channel Blockers | CV200 |
|  | Antimicrobials | AM000 |
|  | Proton Pump Inhibitors | A02BC |
|  | Immunosuppressants | L04A |
|  | Laxatives | GA200 |
