# Supplementary material for: SGLT2 inhibitors, GLP-1 RAs, and DPP4 inhibitors and the risk of hypomagnesemia in type 2 diabetes: A target trial emulation
Source: PLoS Med. 2026 Mar 6;23(3):e1004968. doi: 10.1371/journal.pmed.1004968 (PMC12987583; doi:10.1371/journal.pmed.1004968)
Supplement: S7 Table — (DOCX) [file pmed.1004968.s009.docx]

**S7 Table**. R code for running propensity score matching and Cox proportional hazards model analysis.

## Required packages

library(survival) # for coxph(), Surv()

library(MatchIt) # for propensity score matching

# time: event or censoring time

# status: event indicator (1 = event occurred, 0 = censored)

# treat: treatment/exposure indicator (1 = exposed, 0 = unexposed)

# covariates such as age, sex, body mass index, laboratory values, comorbidity, etc.

## 1) Estimate propensity scores and perform nearest-neighbor matching

m.out <- matchit(

formula = treat ~ age + sex + laboratory values + comorbidities + comedications, # specify covariates for PS model

data = data,

method = "nearest”, # nearest-neighbor matching

ratio = 1, # 1:1 matching

caliper = 0.1, # matching distance

replace = FALSE # without replacement

)

## 2) Extract the matched sample

m.data <- match.data(m.out)

## 3) Fit the Cox proportional hazards model on the matched sample

For the visualization of Kaplan-Meier curves, the ggbreak package was employed to implement axis breaks and effectively utilize plotting space (Xu S, Chen M, Feng T, Zhan L, Zhou L, Yu G. Use ggbreak to Effectively Utilize Plotting Space to Deal With Large Datasets and Outliers. Front Genet. 2021;12:774846).
