# Supplementary material for: SGLT2 inhibitors, GLP-1 RAs, and DPP4 inhibitors and the risk of hypomagnesemia in type 2 diabetes: A target trial emulation
Source: PLoS Med. 2026 Mar 6;23(3):e1004968. doi: 10.1371/journal.pmed.1004968 (PMC12987583; doi:10.1371/journal.pmed.1004968)
Supplement: S8 Table — (DOCX) [file pmed.1004968.s010.docx]

| **Supplementary Table 8.** Hypomagnesemia risk across different follow-up time intervals after 1:1 propensity-score matching | | | |
| --- | --- | --- | --- |
|  | SGLT2 inhibitors vs.  DPP4 inhibitors [Reference] | GLP-1 RAs vs.  DPP4 inhibitors [Reference] | SGLT2 inhibitors vs.  GLP-1 RAs [Reference] |
|  | HR (95% CI) | HR (95% CI) | HR (95% CI) |
| First year of follow-up | 0.81 (0.80, 0.83) | 0.79 (0.78, 0.81) | 1.04 (1.03, 1.06) |
| Second year of follow-up | 0.89 (0.87, 0.91) | 0.96 (0.94, 0.98) | 0.93 (0.92, 0.95) |
| Third year of follow-up | 0.84 (0.82, 0.86) | 0.95 (0.93, 0.98) | 0.89 (0.87, 0.91) |
| Fourth year of follow-up | 0.79 (0.77, 0.81) | 0.93 (0.91, 0.96) | 0.85 (0.83, 0.88) |
| Fifth year of follow-up | 0.75 (0.73, 0.78) | 0.94 (0.91, 0.96) | 0.81 (0.79, 0.84) |
| CI: confidence intervals, DPP4: dipeptidyl peptidase-4, GLP-1 RA: glucagon-like peptide-1 receptor agonist, HR: hazard ratio, SGLT2: sodium–glucose cotransporter 2 | | | |
