# Supplementary material for: SGLT2 inhibitors, GLP-1 RAs, and DPP4 inhibitors and the risk of hypomagnesemia in type 2 diabetes: A target trial emulation
Source: PLoS Med. 2026 Mar 6;23(3):e1004968. doi: 10.1371/journal.pmed.1004968 (PMC12987583; doi:10.1371/journal.pmed.1004968)
Supplement: S9 Table — (DOCX) [file pmed.1004968.s011.docx]

| **S9 Table.** Hypomagnesemia risk by individual drugs after 1:1 propensity-score matching. | |
| --- | --- |
|  | HR (95% CI) |
| Empagliflozin vs. DPP4 inhibitors | 0.84 (0.83, 0.85) |
| Dapagliflozin vs. DPP4 inhibitors | 0.88 (0.87, 0.90) |
| Canagliflozin vs. DPP4 inhibitors | 0.75 (0.73, 0.78) |
| Ertugliflozin vs. DPP4 inhibitors | 0.91 (0.83, 0.997) |
|  |  |
| Liraglutide vs. DPP4 inhibitors | 0.88 (0.86, 0.89) |
| Semaglutide vs. DPP4 inhibitors | 0.84 (0.82, 0.85) |
| Dulaglutide vs. DPP4 inhibitors | 0.94 (0.93, 0.96) |
| Lixisenatide vs. DPP4 inhibitors | 0.98 (0.90, 1.07) |
| Exenatide vs. DPP4 inhibitors | 0.93 (0.89, 0.97) |
| Albiglutide vs. DPP4 inhibitors | 0.86 (0.75, 0.99) |
