# Supplementary material for: SGLT2 inhibitors, GLP-1 RAs, and DPP4 inhibitors and the risk of hypomagnesemia in type 2 diabetes: A target trial emulation
Source: PLoS Med. 2026 Mar 6;23(3):e1004968. doi: 10.1371/journal.pmed.1004968 (PMC12987583; doi:10.1371/journal.pmed.1004968)
Supplement: S10 Table — (DOCX) [file pmed.1004968.s012.docx]

| **S10 Table.** Results of sensitivity analyses. | |
| --- | --- |
|  | HR (95% CI) |
| **SGLT2 inhibitors vs. DPP4 inhibitors** |  |
| Excluding patients with other disorders of fluid, electrolyte and acid-base balance at baseline | 0.80 (0.79, 0.81) |
| Hypomagnesemia redefined as intravenous administration of magnesium sulphate or magnesium chloride only | 0.93 (0.91, 0.95) |
| Hypomagnesemia redefined as serum Mg^2+^ level <1.8 mg/dL only | 0.79 (0.78, 0.80) |
| Excluding patients who had previously undergone serum magnesium testing at baseline | 0.83 (0.82, 0.84) |
| Excluding patients who switched to or initiated another drug class during follow-up | 0.81 (0.80, 0.82) |
| Hyperkalemia occurrence (positive control outcome) | 0.84 (0.82, 0.86) |
| Appendicitis occurrence (negative control outcome) | 1.03 (0.93, 1.14) |
|  |  |
| **GLP-1 RAs vs. DPP4 inhibitors** |  |
| Excluding patients with other disorders of fluid, electrolyte and acid-base balance at baseline | 0.87 (0.86, 0.88) |
| Hypomagnesemia redefined as intravenous administration of magnesium sulphate or magnesium chloride only | 0.97 (0.96, 0.99) |
| Hypomagnesemia redefined as serum Mg^2+^ level <1.8 mg/dL only | 0.88 (0.86, 0.89) |
| Excluding patients who had previously undergone serum magnesium testing at baseline | 0.91 (0.90, 0.92) |
| Excluding patients who switched to or initiated another drug class during follow-up | 0.86 (0.85, 0.87) |
| Hyperkalemia occurrence (positive control outcome) | 0.91 (0.89, 0.93) |
| Appendicitis occurrence (negative control outcome) | 1.07 (0.96, 1.19) |
|  |  |
| **SGLT2 inhibitors vs. GLP-1 RAs** |  |
| Excluding patients with other disorders of fluid, electrolyte and acid-base balance at baseline | 0.92 (0.90, 0.93) |
| Hypomagnesemia redefined as intravenous administration of magnesium sulphate or magnesium chloride only | 0.95 (0.93, 0.97) |
| Hypomagnesemia redefined as serum Mg^2+^ level <1.8 mg/dL only | 0.92 (0.91, 0.93) |
| Excluding patients who had previously undergone serum magnesium testing at baseline | 0.90 (0.89, 0.91) |
| Excluding patients who switched to or initiated another drug class during follow-up | 0.96 (0.94, 0.97) |
| Hyperkalemia occurrence (positive control outcome) | 0.96 (0.94, 0.98) |
| Appendicitis occurrence (negative control outcome) | 0.97 (0.88, 1.08) |
